# Supplementary material for: A morphology-based machine learning model for scoring epithelial-mesenchymal plasticity using organelle dynamics
Source: Commun Biol. 2025 Dec 10;9:59. doi: 10.1038/s42003-025-09326-8 (PMC12800221; doi:10.1038/s42003-025-09326-8)
Supplement: Supplementary file 1 — SupplementaryInformation [file 42003_2025_9326_MOESM1_ESM.pdf]

A Morphology-Based Machine Learning Model for Scoring Epithelial-Mesenchymal Plasticity using Organelle Dynamics

Justin Slager[1, #], Francesca Gatto[1, #], Benjamin Frey[2], Wenyang Shi[1], Bartlomiej Porebski[3, 4], Jordi Carreras-Puigvert[2, 4], Malgorzata Maria Parniewska[1] and Jonas Fuxe[1, *]

Supplementary Information

**Supplementary Figure 1:**	**page 2**
**Supplementary Figure 2:**	**page 3**
**Supplementary Figure 3:**	**page 4**
**Supplementary Figure 4:**	**page 5**
**Supplementary Figure 5:**	**page 6**
**Supplementary Figure 6:**	**page 7**
**Supplementary Figure 7:**	**page 8**
**Supplementary Figure 8:**	**page 9**
**Supplementary Figure 9:**	**page 10**
**Supplementary Table 4:**	**page 11**
**Supplementary Table 5:**	**page 12**

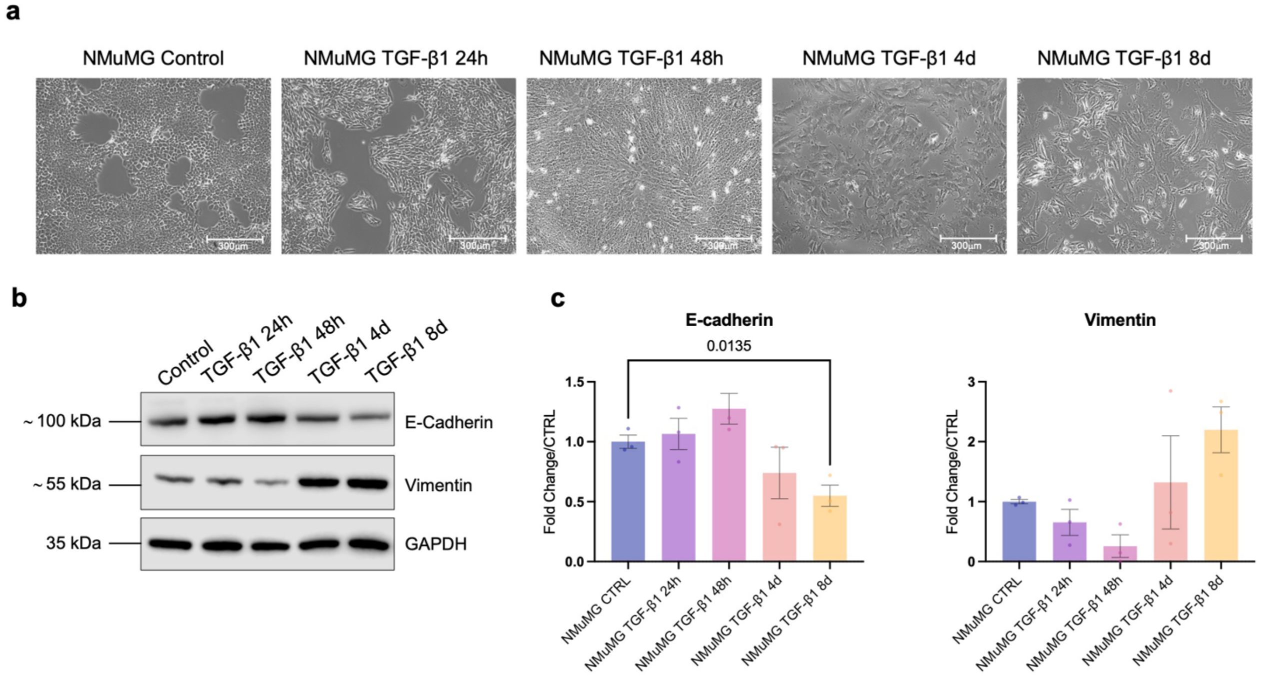

Figure S1. TGF-β1 induces EMT in NMuMG cells. a, Representative brightfield images showing morphological changes of NMuMG cells treated with TGF-β1 over a time course (24h, 48h, 4days, 8days), progressing from an epithelial-like morphology to an elongated, spindle-shaped mesenchymal phenotype. Scale bars = 300μm. **b,** Representative Western blot analysis of EMT markers at different time points following TGF-β1 stimulation in NMuMG cells. **c,** Quantification of Western blot protein expression levels normalized to the control group (NMuMG CTRL), which were cells left untreated. Bars represent mean ± SEM, with numerical p-values indicated for statistically significant comparisons of three independent experiments.

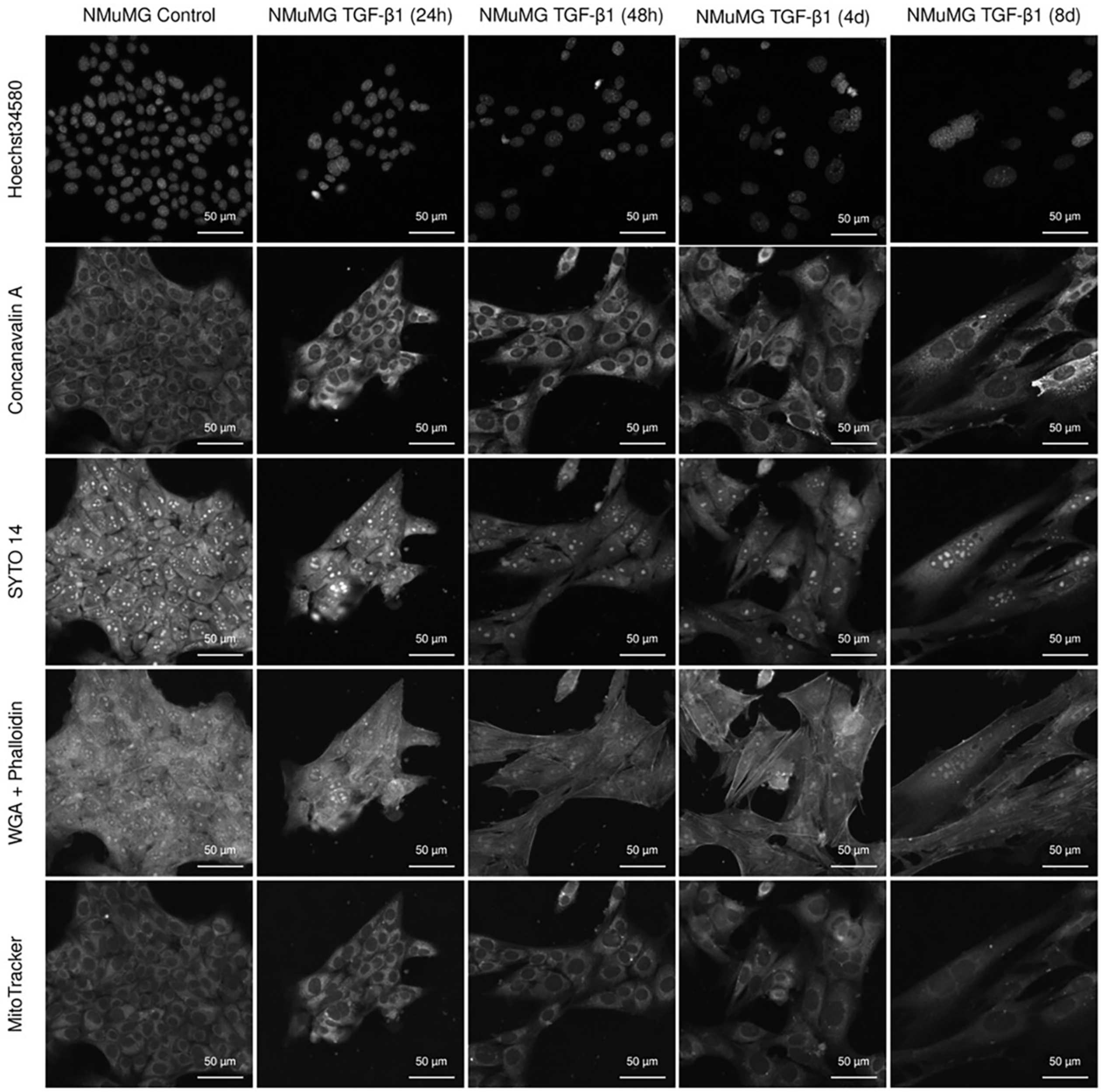

Figure S2. Visualization of organelle dynamics during TGF-β1-induced EMT in NMuMG cells. Representative fluorescent images of NMuMG cells stained with the Cell Painting assay to visualize organelle changes at different stages of TGF-β1-induced EMT. Images were taken on a high-throughput Nikon CrEST X-Light V3 spinning disc imaging system, with a 20x objective. Scale bars represent 50 µm. The intensity values of all images displayed were adjusted for enhanced visualization, adjustments were equalized for fair image comparison.

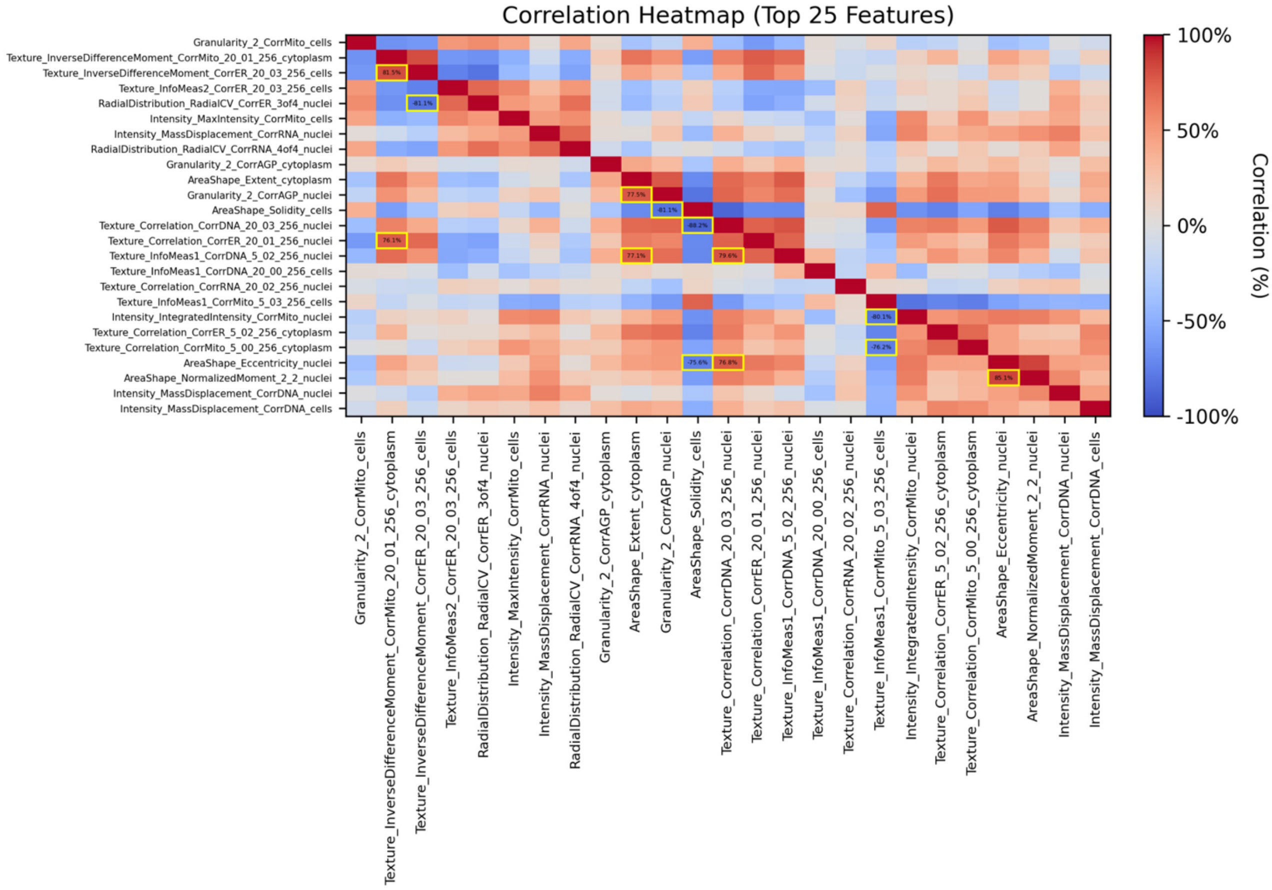

Figure S3. Refinement of data by removal of redundant features. A correlation heatmap based on Spearmans ranking was calculated and plotted. Highly correlated (absolute value of 75%) features (highlighted by a yellow box) were removed due to redundancy.

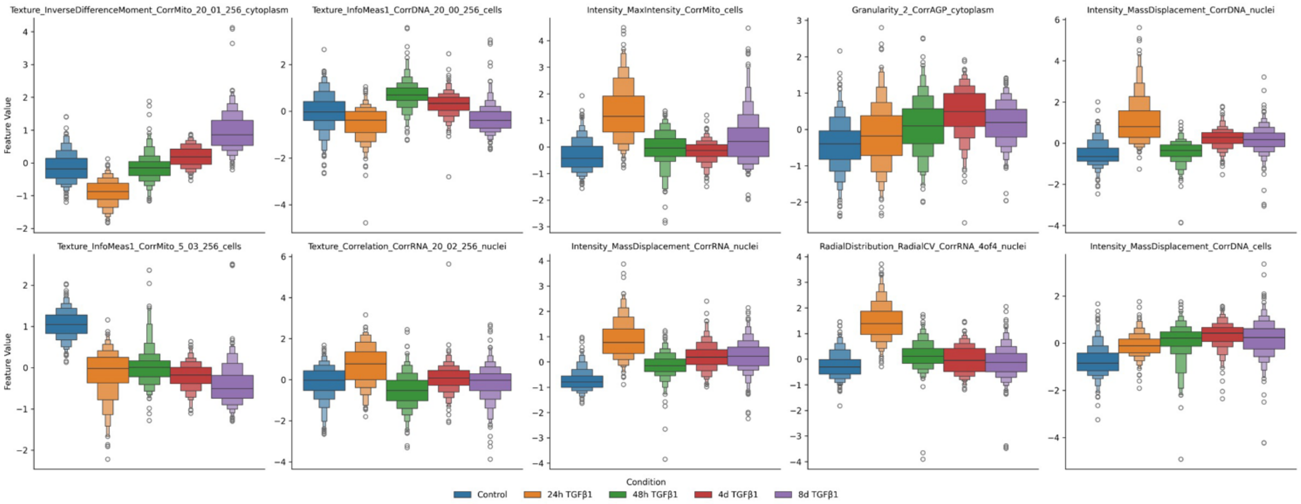

Figure S4. Distribution of top 6-15 features across different stages of TGF-β1-induced EMT. Probability distribution of model-predicted conditions for aggregated profiles in individual wells. Each subplot represents all the wells containing the same experimental condition. The x-axis denotes well IDs, and the y-axis represents the probability (0 to 1) assigned by the model for each condition. Error bars indicate variability in the predictions. (N=9 per well)

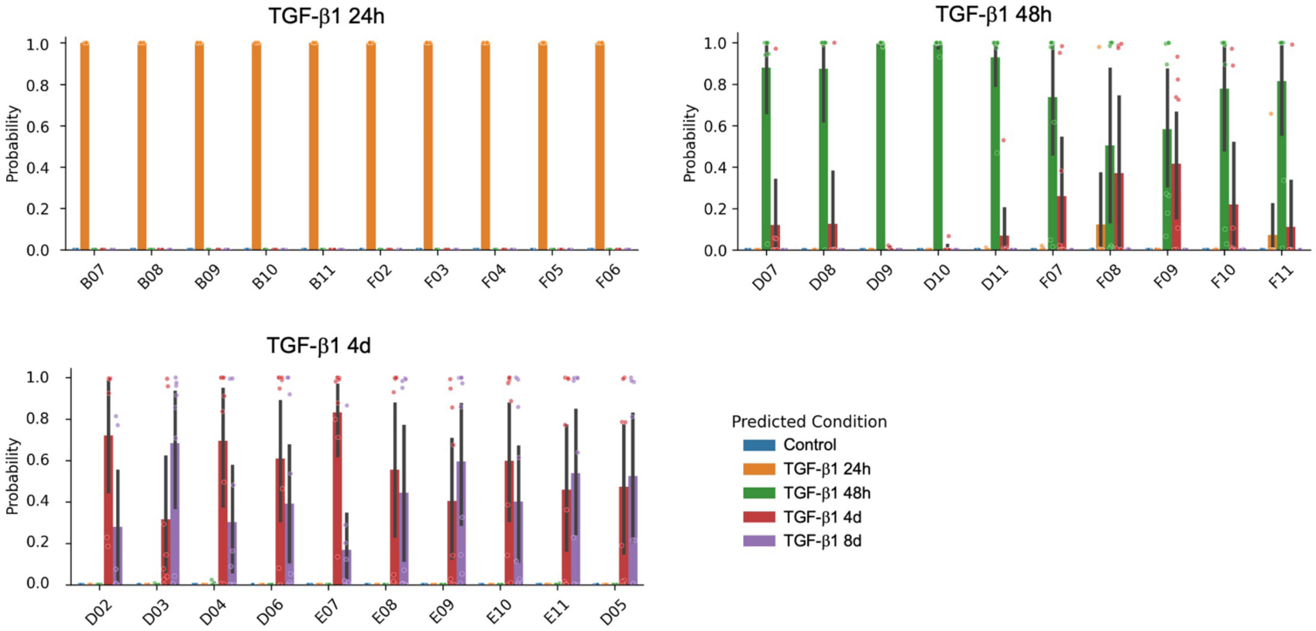

Figure S5. Probability distribution of model-predicted conditions for aggregated profiles in individual wells. Each subplot represents all the wells containing the same experimental condition. The x-axis denotes well IDs, and the y-axis represents the probability (0 to 1) assigned by the model for each condition. Bars are color-coded as indicated. Error bars indicate variability (95% confidence interval) in the predictions. (N=9 per well)

a

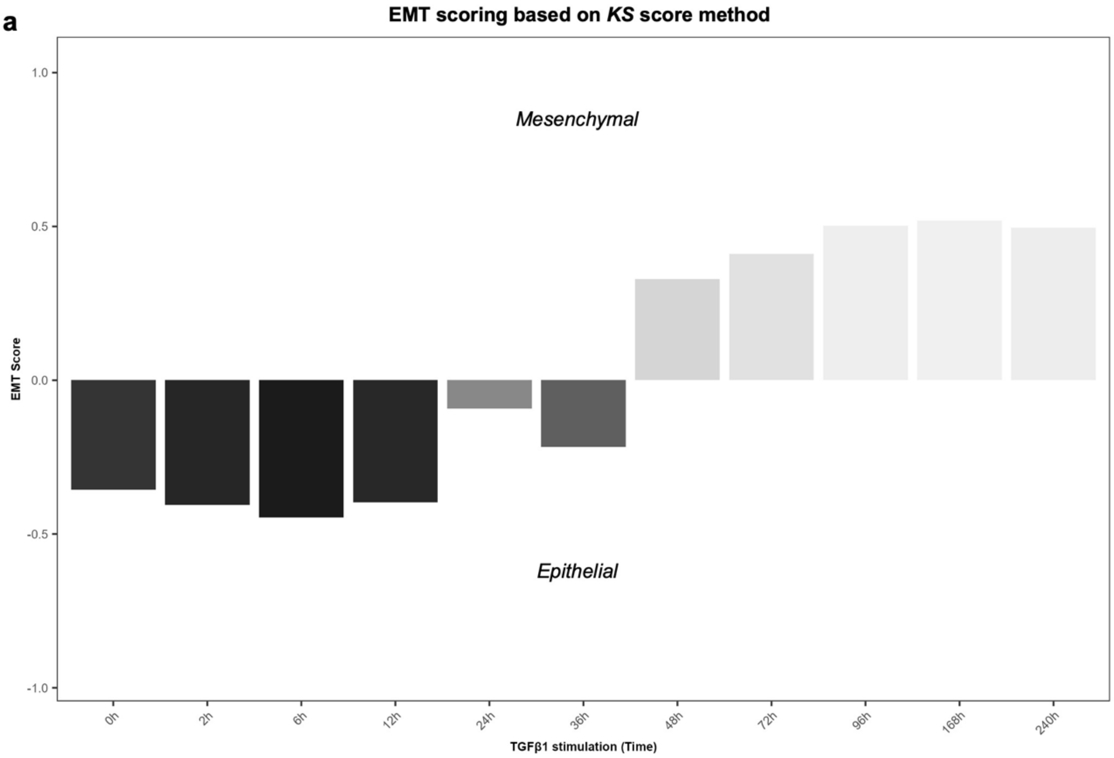

b

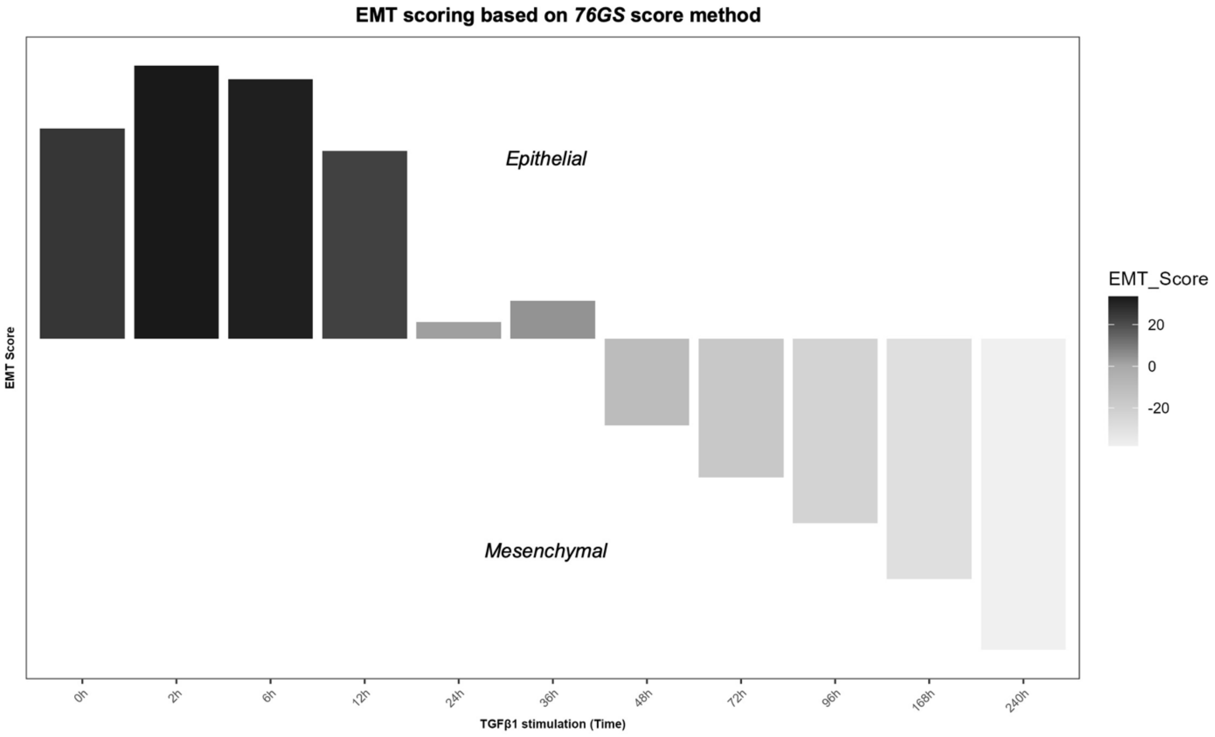

Figure S6. EMT scores based on gene expression analysis. a, b Scoring of EMT at different stages of TGF-β1-induced EMT in NMuMG cells by gene expression methods derived from Chakraborty et al. (2020) (**a**, KSScore) and the weighted sum of 76 EMT-related genes correlated to CDH1 (E-Cadherin) (**b**, 76GS score).

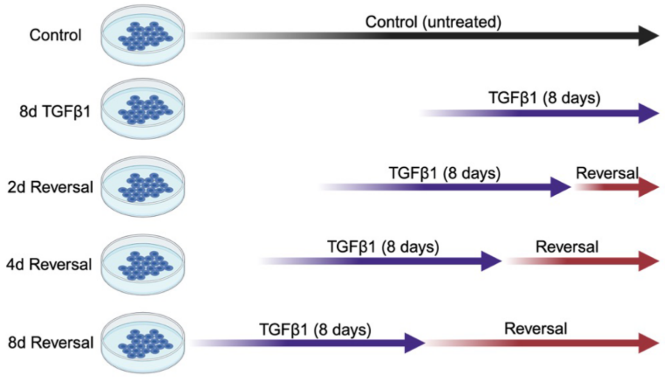

Figure S7. Schematic illustration of the EMT reversal experiment. NMuMG cells were either unstimulated (control) or stimulated with TGF-β1 for 8 days, followed by withdrawal TGF-β1 from the culture medium for 2, 4, or 8 days, to study reversal of EMT. Image created with Biorender.com.

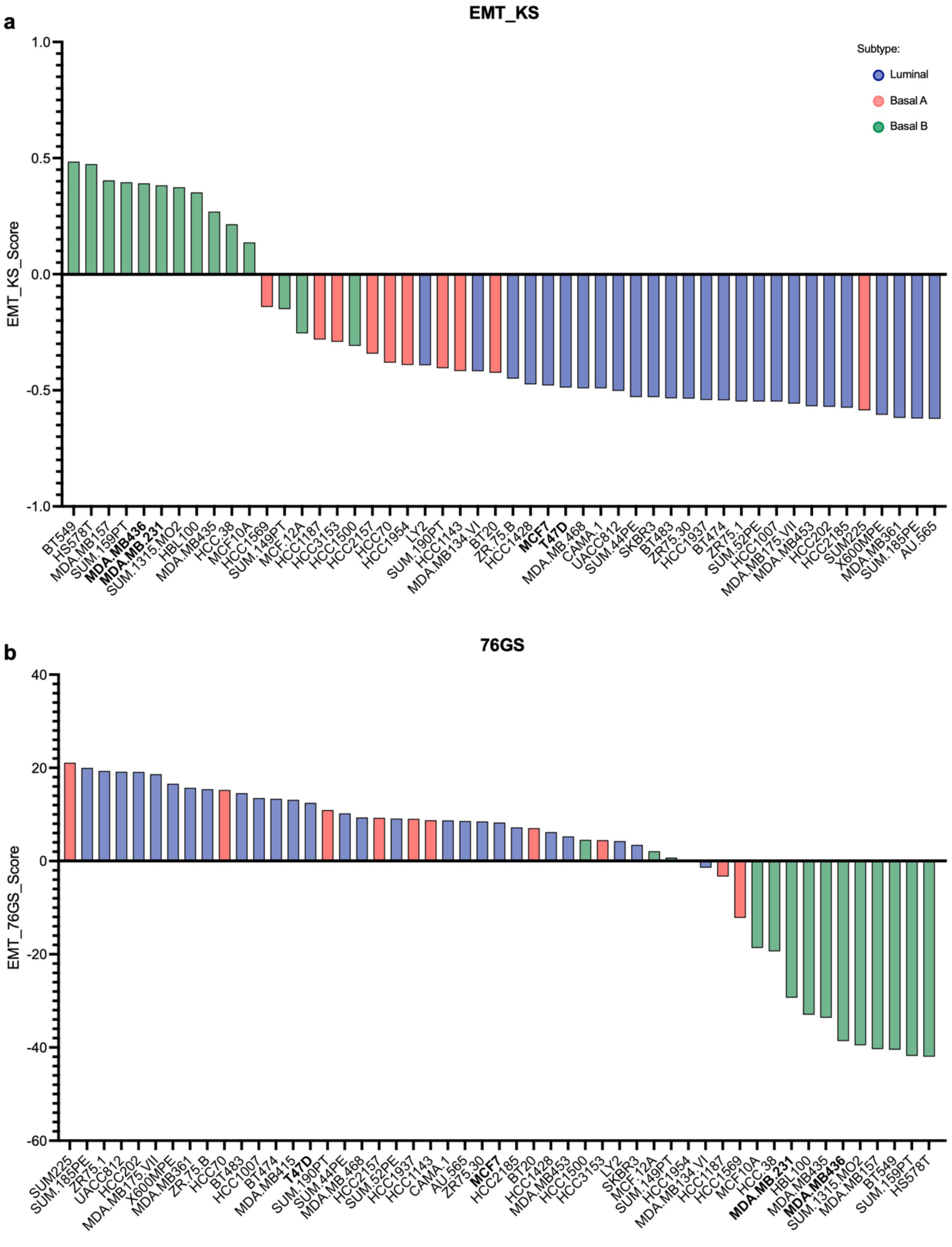

Figure S8. EMT scores across breast cancer cell lines using transcriptional scoring methods. a, EMT scores of 52 human breast cancer cell lines from the GOBO database were calculated using the KSScore method. Cell lines are ranked along the EMT spectrum, with mesenchymal and epithelial phenotypes represented by positive and negative probabilities, respectively. Subtypes classification (Basal A, Basal B, and Luminal) were adopted from the GOBO database and are color-coded for visualization. Breast cancer cell lines available in-house and subjected to EMT scoring are displayed in bold font. **b,** EMT scores of the same 52 cell lines were calculated using the 76GS scoring method. Results are consistent with the KSScore method, further validating the classification of epithelial and mesenchymal phenotypes across different breast cancer cell subtypes.

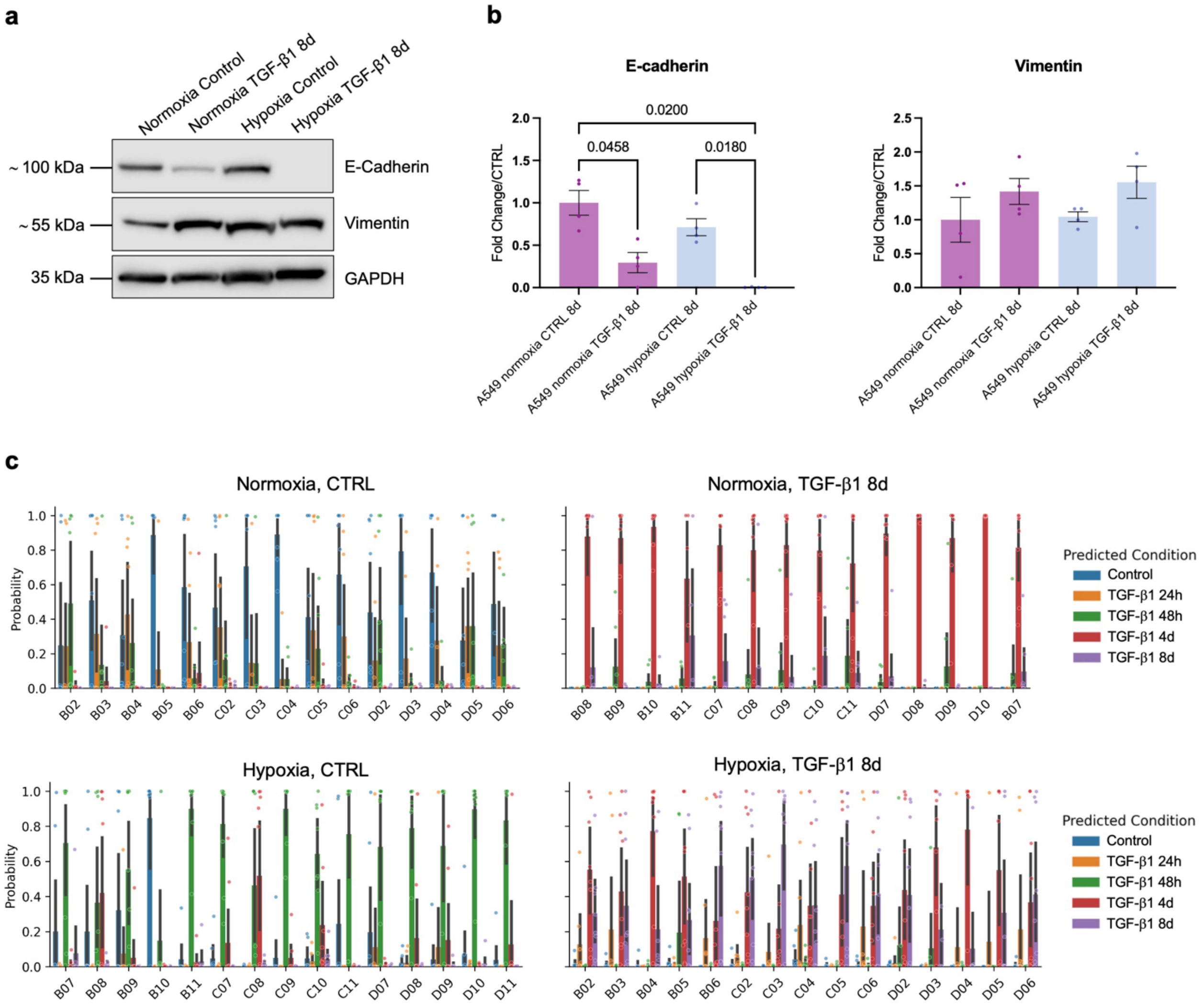

Figure S9. Hypoxia enhances TGF-β1-induced EMT on A549 cells. a, Representative Western blot analysis of EMT markers E-cadherin and vimentin in A549 cells normoxic and hypoxic conditions, with or without TGF-β1 stimulation for 8d. **b**, Quantification of Western blot protein expression levels normalized to the control group (A549 normoxia CTRL), which were cells left untreated. Bars represent mean ± SEM, with numerical p-values indicated for statistically significant comparisons of three independent experiments. **c**, Probability distribution for aggregated profiles in individual wells of A549 cells in normoxic and hypoxic conditions, either left untreated or stimulated with TGF-β1 for 8d. The x-axis denotes well IDs, and the y-axis represents the probability (0 to 1) of cells in each well to belong to the predicted condition. Bars are color-coded as indicated. Error bars indicate variability (95% confidence interval) in the predictions (N=9 per well).

Supplementary Table 4: Image-iT^{TM} Cell Painting Kit dilution scheme.

Dye (stock concentration)	Dilution from stock solution	Final concentration	Per 96-well plate (4 mL)
**Hoechst 34580 (10 mg/mL)**	1:20000	0.5 µg/mL	0.2 µL
**Concanavalin A, Alexa Fluor 488 (5mg/ml)**	1:1000	5 µg/mL	4 µL
**SYTO 14 stain (5 mM)**	1:833	6 µM	4.8 µL
**Wheat Germ Agglutinin, Alexa Fluor 555 (1 mg/ml)**	1:666	1.5 µg/mL	6 µL
**Alexa Fluor 568 Phalloidin (66 µM)**	1:8000	8.25 nM	0.5 µL

Supplementary Table 5: Image-iT^{TM} Cell Painting Kit microscopy settings.

Dye	Laser	Organelle/cellular component
**Hoechst 34580**	405	Nucleus
**Concanavalin A/Alexa Fluor488**	477	Endoplasmic reticulum
**SYTO 14 green, fluorescent stain**	518	Nucleoli, cytoplasmic RNA
**Phalloidin Alexa Fluor 568**	545	F-actin cytoskeleton
**Wheat germ agglutinin Alexa Fluor 555**	545	Golgi, plasma membrane
**MitoTracker Deep Red**	637	Mitochondria
