# Supplementary material for: A morphology-based machine learning model for scoring epithelial-mesenchymal plasticity using organelle dynamics
Source: Commun Biol. 2025 Dec 10;9:59. doi: 10.1038/s42003-025-09326-8 (PMC12800221; doi:10.1038/s42003-025-09326-8)
Supplement: Supplementary file 2 — Description of Additional Supplementary Files [file 42003_2025_9326_MOESM2_ESM.pdf]

Description of Additional Supplementary Files

File name: Supplementary Table 1

Description: List of 2,608 morphological output features.

File name: Supplementary Table 2

Description: Classification report.

File name: Supplementary Table 3

Description: Plate layouts.
