# Supplementary material for: A morphology-based machine learning model for scoring epithelial-mesenchymal plasticity using organelle dynamics
Source: Commun Biol. 2025 Dec 10;9:59. doi: 10.1038/s42003-025-09326-8 (PMC12800221; doi:10.1038/s42003-025-09326-8)
Supplement: Supplementary file 6 — Reporting Summary [file 42003_2025_9326_MOESM6_ESM.pdf]

nature portfolio

Corresponding author(s): *Double-anonymous peer review submissions: write DAPR and your manuscript number here instead of author names.*

Last updated by author(s): *YYYY-MM-DD*

Reporting Summary

Nature Portfolio wishes to improve the reproducibility of the work that we publish. This form provides structure for consistency and transparency in reporting. For further information on Nature Portfolio policies, see our Editorial Policies and the Editorial Policy Checklist.

Statistics

For all statistical analyses, confirm that the following items are present in the figure legend, table legend, main text, or Methods section.

n/a	Confirmed	
☐	☒	The exact sample size (*n*) for each experimental group/condition, given as a discrete number and unit of measurement
☐	☒	A statement on whether measurements were taken from distinct samples or whether the same sample was measured repeatedly
☐	☒	The statistical test(s) used AND whether they are one- or two-sided<br>*Only common tests should be described solely by name; describe more complex techniques in the Methods section.*
☐	☒	A description of all covariates tested
☐	☒	A description of any assumptions or corrections, such as tests of normality and adjustment for multiple comparisons
☐	☒	A full description of the statistical parameters including central tendency (e.g. means) or other basic estimates (e.g. regression coefficient) AND variation (e.g. standard deviation) or associated estimates of uncertainty (e.g. confidence intervals)
☐	☒	For null hypothesis testing, the test statistic (e.g. *F*, *t*, *r*) with confidence intervals, effect sizes, degrees of freedom and *P* value noted<br>*Give P values as exact values whenever suitable.*
☒	☐	For Bayesian analysis, information on the choice of priors and Markov chain Monte Carlo settings
☒	☐	For hierarchical and complex designs, identification of the appropriate level for tests and full reporting of outcomes
☒	☐	Estimates of effect sizes (e.g. Cohen's *d*, Pearson's *r*), indicating how they were calculated

Our web collection on statistics for biologists contains articles on many of the points above.

Software and code

Policy information about availability of computer code

Data collection	NIS elements software (version AR 5.30.0 2)
Data analysis	CellProfiler (version 4. 2.5); Python (version 3.1 2.4); R software (4.4.0); GraphPad Prism (version 10.4.0)

For manuscripts utilizing custom algorithms or software that are central to the research but not yet described in published literature, software must be made available to editors and reviewers. We strongly encourage code deposition in a community repository (e.g. GitHub). See the Nature Portfolio guidelines for submitting code & software for further information.

Data

Policy information about availability of data

All manuscripts must include a data availability statement. This statement should provide the following information, where applicable:

- Accession codes, unique identifiers, or web links for publicly available datasets
- A description of any restrictions on data availability
- For clinical datasets or third party data, please ensure that the statement adheres to our policy

Image data, numerical data and uncropped western blot data used for analyses in the manuscript are available at https://figshare.scilifelab.se/account/home#/projects/234446

Research involving human participants, their data, or biological material

Policy information about studies with human participants or human data. See also policy information about sex, gender (identity/presentation), and sexual orientation and race, ethnicity and racism.

Reporting on sex and gender	NA
Reporting on race, ethnicity, or other socially relevant groupings	NA
Population characteristics	NA
Recruitment	NA
Ethics oversight	NA

Note that full information on the approval of the study protocol must also be provided in the manuscript.

Field-specific reporting

Please select the one below that is the best fit for your research. If you are not sure, read the appropriate sections before making your selection.

☒ Life sciences ☐ Behavioural & social sciences ☐ Ecological, evolutionary & environmental sciences

For a reference copy of the document with all sections, see nature.com/documents/nr-reporting-summary-flat.pdf

Life sciences study design

All studies must disclose on these points even when the disclosure is negative.

Sample size	The multi-level sampling structure was intentionally designed to capture both biological and technical variability. Biological variability is accounted for by using multiple independent plates, while technical variability is addressed by including multiple wells and images per well. Due to our sample size design the statistical power of our study allowed for detection of subtle phenotypic differences with high confidence.
Data exclusions	Aggregated data from the three plates were preprocessed to remove missing values and normalized per plate using robust scaling based on the median and interquartile range (IQR) per feature across conditions. Feature selection was applied to remove highly correlated (>0.9), low-variance (<0.01), and meta data-rich columns using Pycytominer (version 1.1.0). A blacklist was applied to remove specific features from the normalized and aggregated dataset. Features were excluded based on previous experience and predefined patterns associated with irrelevant or redundant measurements.
Replication	All attempts to replicate the findings were succesful
Randomization	Treatment allocation was done to ensure that replicates were assigned different spots in the 96-well plates. For the in silica analysis, the dataset was randomly divided into training and test sets to enable rigorous training and evaluation of multiple classifiers.
Blinding	The investigator seeded cells in the wells but the machine learning was performed using label-blinding.

Reporting for specific materials, systems and methods

We require information from authors about some types of materials, experimental systems and methods used in many studies. Here, indicate whether each material, system or method listed is relevant to your study. If you are not sure if a list item applies to your research, read the appropriate section before selecting a response.

Materials & experimental systems

n/a	Involved in the study	
☐	☒	Antibodies
☐	☒	Eukaryotic cell lines
☒	☐	Palaeontology and archaeology
☒	☐	Animals and other organisms
☒	☐	Clinical data
☒	☐	Dual use research of concern
☒	☐	Plants

Methods

n/a	Involved in the study	
☒	☐	ChIP-seq
☒	☐	Flow cytometry
☒	☐	MRI-based neuroimaging

Antibodies

Antibodies used	Primary antibodies used were: E-cadherin (#24E10, Cell Signaling Technology, Leiden, The Netherlands); Vimentin (#5741, Cell Signaling Technology); GAPDH (#MA5-15738-HRP, Thermofisher). Secondary antibodies used were: HRP-conjugated goat anti-rabbit and anti-mouse IgG (#7074, #7076 Cell Signaling Technology).
Validation	E-cadherin #24E10; validated for WB and cross-reactive with Human and Mouse species (https://www.cellsignal.com/products/primary-antibodies/e-cadherin-24e10-rabbit-mab/3195)<br>Vimentin #5741; validated for WB and cross-reactive with Human and Mouse species https://www.cellsignal.com/products/primary-antibodies/vimentin-d21h3-xp-rabbit-mab/5741<br>GAPDH, #MA5-15738-HRP; validated for WB and cross-reactive with Human and Mouse species (https://www.thermofisher.com/antibody/product/GAPDH-Loading-Control-Antibody-clone-GA1R-Monoclonal/MA5-15738-HRP)<br>Goat anti-rabbit and anti-mouse IgG (#7074, #7076 Cell Signaling Technology). Both validated for validated for WB (https://www.cellsignal.com/products/secondary-antibodies/anti-rabbit-igg-hrp-linked-antibody/7074; https://www.cellsignal.com/products/secondary-antibodies/anti-mouse-igg-hrp-linked-antibody/7076)

Eukaryotic cell lines

Policy information about cell lines and Sex and Gender in Research

Cell line source(s)	ATCC
Authentication	ATCC
Mycoplasma contamination	Regularly checked at Eurofins
Commonly misidentified lines (See ICLAC register)	None of the used ones

Plants

Seed stocks	NA
Novel plant genotypes	NA
Authentication	NA
